# Microbial Community and Functional Analysis of Regionally Produced Traditional Korean Grain Vinegar

**DOI:** 10.3390/microorganisms13061308

**Published:** 2025-06-04

**Authors:** Su Jeong Lee, Sun Hee Kim, Hee-Min Gwon, Jinju Park

**Affiliations:** Fermented and Processed Food Research Division, Department of Food Sciences, NICS, RDA, Wanju 55365, Republic of Korea; leesooj@korea.kr (S.J.L.); sunheekim00@korea.kr (S.H.K.); vitamin89@korea.kr (H.-M.G.)

**Keywords:** vinegar, grain vinegar, fermentation, microbial community

## Abstract

This study investigated changes in microbial communities and functional components during the fermentation of traditional Korean grain vinegars collected from various regions as well as the correlations among these components. Microbial community analysis revealed that *Lactobacillus acetotolerans* was the dominant microorganism, while *Acetobacter pasteurianus* numbers gradually increased during fermentation, playing a key role in acetic acid production. *L. acetotolerans*, known to thrive in acidic environments, contributed to increasing the acidity of the vinegar and enhanced its preservative properties. The rise in the levels of organic acids, particularly acetic acid, was influenced by the activity of these microorganisms. Additionally, the production of free amino acids, such as alanine, was influenced by interactions between the fermentation medium and microbial communities, significantly contributing to the vinegar’s sweetness. Volatile flavor compounds exhibited considerable diversity due to changes in microbial communities driven by raw-material differences. In particular, five-grain vinegar (YO) tended to generate more complex and intense flavor compounds, with uniformly elevated levels of aldehydes, acids, and ketones. These findings suggest that raw-material selection and fermentation conditions significantly influence the flavor and functional properties of grain vinegars, providing valuable foundational data for improving vinegar production processes to enhance flavor and functionality.

## 1. Introduction

Vinegar is a fermented food widely consumed around the world, with a tradition that dates back to ancient times [1]. Due to its unique taste, aroma, and beneficial health effects, vinegar has long been used as a condiment and preservative. In countries such as Korea, Japan, China, and Spain, traditional vinegar production methods involve fermenting grain- or fruit-based raw materials [2]. Various types of vinegar exist depending on the country or region. The flavor, aroma, and health benefits of vinegar are largely determined by the microbial composition, raw materials, and technical methods used in the fermentation process [3,4,5].

The traditional vinegar fermentation process is divided into two stages. The first stage, alcohol fermentation, is the process by which yeast converts sugars into alcohol. The second stage, acetic acid fermentation, is the process through which acetic acid bacteria convert this alcohol into acetic acid [6]. Thus, vinegar fermentation is driven by a range of microorganisms that affect the product’s final quality. Previous studies have shown that identifying changes in microbial communities during vinegar fermentation can provide important clues for improving vinegar quality or discovering new health benefits [7,8,9]. Microbial community analyses have been conducted on various fermentation systems, ranging from traditional to modern, advanced methods. These analyses have helped identify key microorganisms involved in vinegar fermentation, thereby improving fermentation efficiency [4,10].

Traditional fermented vinegars in Korea, which undergo natural fermentation processes, are mainly grain vinegars made from rice and barley and fruit vinegars made from apples and pears. However, with the advancement of brewing techniques for grain-based liquors, a food culture centered around grain vinegar has emerged [11,12]. Since the 1990s, there has been an increased awareness of health promotion and dietary habits, leading to the growing popularity of traditional vinegars, which are considered to have superior functional properties compared to commercial vinegars [13]. In natural fermentation processes, which do not involve starter cultures, flavors and functional components are generated by the activities of undefined microbial communities. Therefore, it is necessary to identify these microorganisms [14,15].

Therefore, in this study, traditional Korean grain vinegars were collected from different regions, and the changes in microbial communities and the correlations of functional components over the course of fermentation were monitored. Based on this data, we aimed to identify the dominant microorganisms as well as the growth of harmful microorganisms during the fermentation process and understand the impact of raw materials and environmental factors on microbial growth. The results of this study are expected to provide foundational insights into the relationships between raw materials, microbial communities, and functional compounds in traditional grain vinegars. These findings may guide the optimization of fermentation conditions, the standardization of production processes, and the development of high-quality, health-promoting vinegars, thereby contributing to the sustainable growth of the vinegar industry.

## 2. Materials and Methods

### 2.1. Collection of Grain Vinegars According to Manufacturing Environment

Four types of traditional Korean grain vinegars were selected from different regions. Information regarding raw materials, manufacturing processes, and fermentation conditions used by each producer was investigated. Samples were collected after 0, 10, and 20 days of acetic acid fermentation to analyze microbial communities and fermentation products.

### 2.2. Acidity

A 1 mL sample was collected and diluted 10 times with 9 mL of distilled water. Following the addition of 2–3 drops of 1% phenolphthalein indicator, the sample was titrated with 0.1N NaOH solution to a final pH of 8.35–8.40. The acidity was calculated and expressed as acetic acid (%).

### 2.3. Microbial Community

To analyze the microbial communities of grain vinegars produced using different manufacturing processes, high-quality DNA was obtained through extraction from each sample, with QC ensuring good quality. A library was constructed from the obtained DNA to create clusters for sequencing. The sequencing was carried out using the Illumina HiSeq platform. The raw data from the sequencing were analyzed through shotgun metagenome analysis to identify the microbial communities in the collected grain vinegars [16,17]. The Illumina HiSeq raw data, after being sequenced, were classified by sample using the index sequences, and paired-end FASTQ files were generated. The preprocessed data were analyzed using MetaPhlAn4 (v4.0.0), which includes around 1 million microorganisms from NCBI reference genomes and species-level genomic groups. The read counts were mapped to specific marker genes of microbial species using Bowtie2, and species abundance was calculated based on the average number of mapped reads. Species with a marker gene mapping ratio of less than 33% were considered absent and thus excluded [18]. To assess the species diversity and evenness of the microbial community, alpha diversity (richness, Shannon, and Simpson) was calculated based on species abundance using the ‘calculate diversity. R’ script from MetaPhlAn4.

### 2.4. Organic Acids

A total of 10 mL of each sample was filtered through a 0.2 μm membrane filter (Millipore Co., Burlington, MA, USA) and analyzed using a two-pump HPLC system (Shimadzu Co., Kyoto, Japan) via a post-column method. The column used was TSK gel ODS-100 V (5 μm, 4.6 mm × 250 mm), and the mobile phase was 8 mM perchloric acid (pH 2.10). The injection volume was 10 μL, and the flow rate was set to 1.0 mL/min. Detection was performed at 440 nm [19].

### 2.5. Free Amino Acids

To extract free amino acids, 1–2 g of each sample was mixed with 15 times the volume of distilled water and stirred at room temperature for 1 h. The mixture was then left to stand at 4 °C for 30 min, after which the supernatant was filtered through a 0.2 µm syringe filter for use. A 10 μL aliquot of the filtrate was transferred to a vial, and 70 μL of Borate buffer (I) (AccQ-FlourTM Reagent Kit, Waters Co., Milford, MA, USA) and 20 μL of AccQ Flour reagent (AccQ-FlourTM Reagent Kit, Waters Co.) were added. The solution was vortexed, left to stand for 1 min, heated at 55 °C for 10 min, and then allowed to cool to room temperature for use as the test solution. The derivatized test solution was analyzed using HPLC (Alliance 2695, Waters, Milford, MA, USA). The column used was Waters AccQ Tag (3.9 × 150 mm), and the mobile phase was composed of aqueous buffer and 60% ACN. The injection volume was 10 μL, and the flow rate was set to 1.0 mL/min. Detection was performed across the wavelength range of 250–395 nm, as described in [20], to accommodate the varying absorbance maxima of individual amino acids.

### 2.6. Volatile Flavor Components

For the analysis of volatile flavor compounds, a 1 mL aliquot of a 100-fold-diluted sample and 1 mL of tertiary distilled water containing the internal standard were placed in a 20 mL vial and stirred at 550 rpm for 20 min at 50 °C. The sample and headspace were then allowed to reach equilibrium, and volatile compounds were adsorbed onto an SPME fiber (50/30 µm DVB/CAR/PDMS, Supelco Inc., Bellefonte, PA, USA) for 10 min before analysis. The analysis was performed using a GC-2010 Plus and GCMS-TQ 8030 (Shimazu, Tokyo, Japan) equipped with a DB-WAX column (30 mm × 0.25 mm i.d., 0.25 μm film thickness, J&W Scientific, Folsom, CA, USA). Helium was used as the carrier gas at a flow rate of 1 mL/min. The injector temperature was set to 230 °C. The oven temperature program was designed to facilitate the analysis of highly volatile components in vinegar: the temperature was initially maintained at 40 °C for 3 min, increased to 90 °C at a rate of 5 °C/min, further increased to 230 °C at a rate of 19 °C/min, and held at this temperature for 5 min. The ion source temperature was set to 200 °C, and the interface temperature was maintained at 250 °C. The detector voltage was set to 0.1 kV. Volatile compounds were identified using the NIST 11 and Wiley 9 mass-spectral libraries [21].

### 2.7. Statistical Analysis

The experiments were conducted in triplicate, and the mean values are reported. The results were statistically analyzed using one-way analysis of variance (ANOVA) with Duncan’s test (*p* < 0.05) using SPSS (ver. 27.0, SPSS Inc., Chicago, IL, USA). Partial least squares discriminant analysis (PLS-DA) was performed to identify volatile compounds contributing to the separation among samples. Furthermore, volatile compounds that significantly contributed to the differences among groups (*p* < 0.05) were visualized using a heat map generated in R-project (v4.2.0), employing the pheatmap package. Hierarchical clustering was conducted based on Euclidean distance and complete linkage, which are the default settings of the package.

## 3. Results

### 3.1. Analysis of Grain Vinegar According to Manufacturing Environment

**Figure 1:**
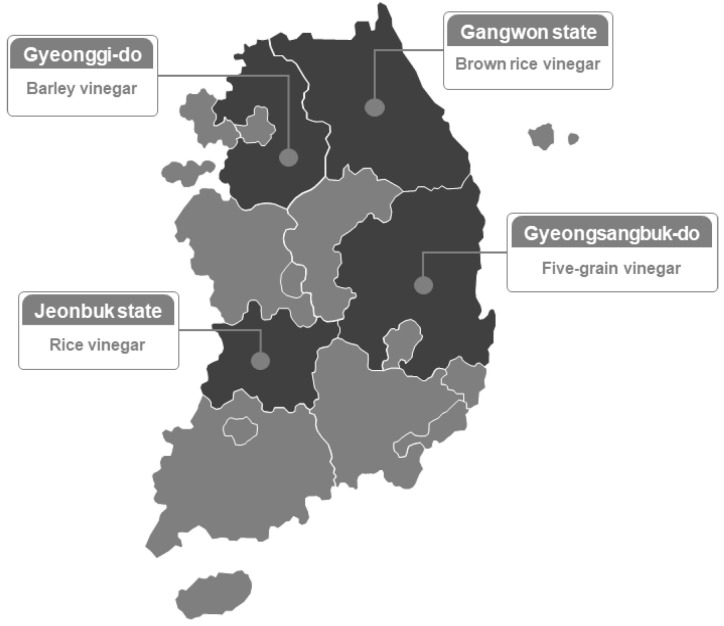
Collection of grain vinegars according to region in Korea.

In this study, four traditional vinegar production companies in Korea were selected, and four types of grain vinegars were collected and analyzed. The regions chosen for this study were Gangwon state, Gyeonggi-do, Jeonbuk state, and Gyeongsangbuk-do. The raw materials used in the production of grain vinegars were brown rice, barley, rice, and a five-grain mixture (brown rice, barley, sorghum, millet, and foxtail millet) in the respective regional order (Figure 1). All four vinegar production processes were conducted without commercial starter cultures. Instead, each manufacturer used previously fermented and stored vinegar as a starter, adding it at approximately 10% (*v*/*v*) to the alcoholic base to initiate acetic acid fermentation. The fermentation period was standardized at three time points: the start of acetic acid fermentation (day 0), the 10th day, and the end of fermentation (day 20). Sampling was performed after confirming that all the manufacturers followed the same fermentation schedule (Table 1).

The brown rice vinegar (HB) from Gangwon state was fermented in earthenware jars at a temperature of 28–30 °C, with acidity measured at 1.60% at the start of fermentation (HB1) and 4.87% on day 20 (HB3). The barley vinegar (SB) from Gyeonggi-do was fermented in a glass container at 30 °C, starting with 1.41% acidity (SB1) on day 0, increasing to 2.51% on day 10 (SB2), and finishing at 6.83% on day 20 (SB3). The rice vinegar (JR) from Jeonbuk state was fermented in earthenware jars at 30 °C, with acidity measured at 1.22% on day 0 (JR1), 2.52% on day 10 (JR2), and 5.43% on day 20 (JR3). This acidity range is consistent with reported data indicating that commercially available grain vinegars have an acidity of between 4% and 6%, confirming sufficient acetic acid production and validating the appropriateness of the fermentation time points [22,23]. The five-grain vinegar (YO) from Gyeongsangbuk-do was fermented in a stainless-steel container at 30 °C. Its initial acidity was determined to be 8.54% on day 0 (YO1), which is very high compared to the other samples. This relatively high initial acidity may be due to either a high inoculation ratio of the finished vinegar or the use of vinegar with a high acetic acid concentration as the starter. On day 10, the acidity increased slightly to 9.53% (YO2), with the final value being 14.67% on day 20 (YO3), a relatively high acidity level. Compared to the initial acidity, the final acidity of HB, SB, and JR increased approximately 3–5 fold, while that of YO increased only about 1.7-fold. This difference is likely due to the manufacturing method, where the fermentation mash is inoculated with prepared vinegar rather than using a starter culture. The varying proportions of fermentation mash and seed vinegar may account for these differences in acidity levels.

The type of vessel used in vinegar production can significantly influence the fermentation environment, thereby affecting the acidity, functional components, and microbial community structure of the final product. Earthenware vessels, due to their porous nature, facilitate effective air and moisture exchange, which can enhance the activity of aerobic microorganisms such as acetic acid bacteria (*Acetobacter* spp.). Fermentation in earthenware vessels has been reported to support a more balanced microbial community, leading to increased acidity and higher concentrations of organic acids [24]. In contrast, glass and stainless-steel vessels, while resistant to external contamination, provide limited temperature and humidity control, which may negatively affect microbial growth. These findings suggest that the physical properties of fermentation vessels play a crucial role in shaping microbial communities and determining vinegar quality.

### 3.2. Microbial Community

#### 3.2.1. Metagenome-Based Microbial Community Analysis

**Figure 2:**
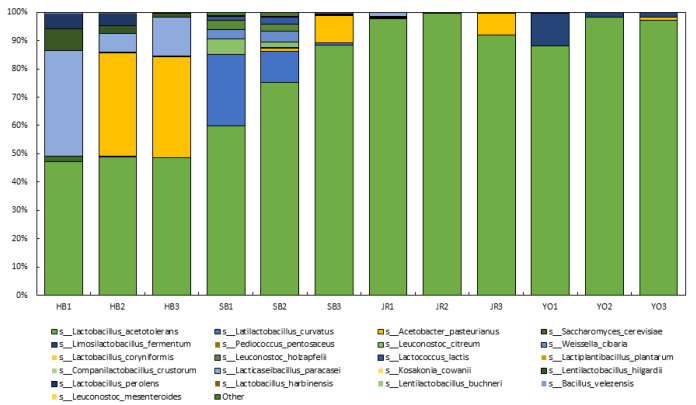
Microbial communities in grain vinegars produced via different fermentation processes across four regions and over three fermentation stages.

Microbial communities are crucial to metabolic processes in both abiotic and biotic systems, including in the generation of secondary metabolites [25,26]. The microbial communities in the four types of grain vinegars collected from different regions were analyzed at different stages of fermentation (Figure 2, Table S1). Libraries were constructed from the DNA extracted from each sample, and, after sequencing, changes in species richness were observed throughout each fermentation process. The microorganisms detected in this study were generally beneficial for vinegar fermentation, contributing positively to the production process. However, *Kosakonia cowanii* was identified as an exception, as it is known to be an opportunistic pathogen associated with plant and human infections [27]. Therefore, its presence raises safety concerns and suggests the need for careful monitoring during vinegar production. Overall, *Lactobacillus acetotolerans* was found to be the dominant species in the vinegars. *L. acetotolerans* accounted for over 40% of the microbial community starting on day 0 of fermentation, indicating that it likely originated from the inoculated starter culture. Its early dominance suggests that it is well adapted to acidic conditions, which may have allowed it to outcompete other microbes. The starter was added at the beginning of fermentation, implying that the species was already present in high abundance within the starter culture. In the rice vinegar (JR), the microbial community established during the early stage of fermentation accounted for more than 90% throughout the fermentation process, peaking at 99.6% on day 10 (JR2). Similarly, the five-grain vinegar (YO) also showed a maximum occupancy of 98.2% on day 10 (YO2). *L. acetotolerans* is known for its resistance to acidic environments such as alcohol and vinegar, and it is associated with increasing the acidity and improving the preservation of food. This finding aligns with previous studies wherein *L. acetotolerans* was isolated from rice vinegar [28].

The presence of *Acetobacter pasteurianus* was notably high in the vinegar samples. In the brown rice vinegar (HB), *A. pasteurianus* showed a rapid increase from 0.1% upon inoculation (HB1) to 36.5% on day 10 (HB2) and remained at 35.5% on day 20 (HB3). In the barley vinegar (SB) and rice vinegar (JR), the levels of *A. pasteurianus* increased to 9.8% and 7.6%, respectively, by day 20. These results are consistent with the acidity measurements, where HB showed a rapid increase in acidity on day 10, whereas SB and JR exhibited higher acidity on day 20. Furthermore, *Lacticaseibacillus paracasei* was observed to decrease in presence from 37.5% on day 0 (HB1) to 6.5% on day 10 (HB2), and then this figure increased again to 13.6% on day 20 (HB3). *L. paracasei* is a strain that thrives in acidic environments, such as in cheese and yogurt. Some strains of *L. paracasei* have been reported to exhibit probiotic potential, including the production of metabolites that may contribute to gut health [29].

At the beginning of fermentation, *Lentilactobacillus hilgardii* in HB, *Latilactobacillus curvatus* in SB, and *Limosilactobacillus fermentum* in YO showed relatively high occupancy rates. These are all types of lactic acid bacteria that consume sugars to produce lactic acid and play a role in inhibiting the growth of pathogens, as reported in previous studies. Their presence in the fermentation mash suggests that these species play an active role during the acetic acid fermentation process [30,31,32]. By identifying the dominant microorganisms in the manufacturing process, we aim to contribute to the development of more-efficient acetic acid fermentation strategies. Additionally, we aim to identify vinegars that harbor beneficial microorganisms and to explore their health-promoting potential.

#### 3.2.2. Diversity Analysis

**Figure 3:**
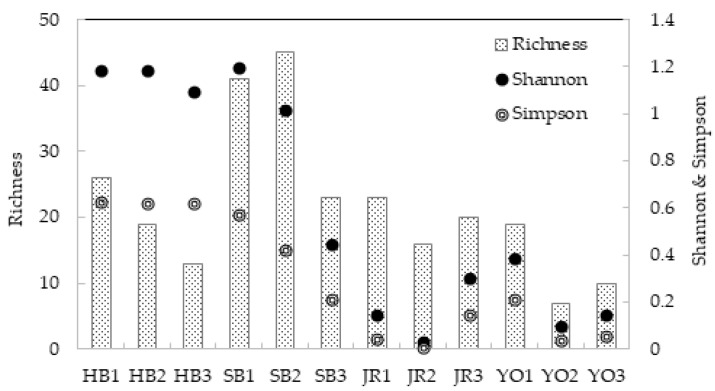
Richness and α-diversity (Shannon and Simpson) in grain vinegars produced via different fermentation processes across four regions and over three fermentation stages.

To assess species diversity, alpha diversity analysis, which is used to measure within-sample diversity, was performed using three indices: species richness (the total number of species within a community); the Shannon index, which reflects diversity by considering both species richness and evenness; and the Simpson index, which estimates the probability of randomly selecting the same species from a given community (Figure 3, Table S2). The total read counts ranged from approximately 74 million to 85 million reads per sample. To minimize potential bias caused by differences in sequencing depth, all the samples were rarefied to a uniform depth of 100,000 reads.

In terms of species richness, the barley vinegar (SB2) exhibited the most diverse microbial community, with 45 species detected. By the end of acetic acid fermentation, 23 species were still present, indicating that this vinegar retained relatively high microbial diversity. The brown rice vinegar (HB) showed a decline in richness, falling from 26 species at the beginning of fermentation (HB1) to 13 species at the end (HB3). The rice vinegar (JR) exhibited a slight reduction, falling from 23 species (JR1) to 20 species (JR3) over the course of fermentation. The five-grain vinegar (YO) exhibited the simplest microbial composition, with 19 species (YO1) at the start and 10 species (YO3) at the end of fermentation. These results suggest that, as acetic acid fermentation progresses, the dominance of key species such as *A. pasteurianus*, essential for vinegar production, leads to the expansion of dominant microbial populations, while the populations of non-dominant species diminish. Consequently, both the Shannon and Simpson indices showed a decreasing trend over time, reflecting a reduction in diversity as fermentation advanced. Among the samples, JR and YO exhibited a slight increase in species richness from day 10 to day 20, although both remained lower than at the start of fermentation. This increase was accompanied by corresponding rises in the Shannon and Simpson diversity indices, suggesting a mild recovery in microbial diversity in the later fermentation stages. This may be attributed to the growth of dominant species such as *A. pasteurianus*, whose abundance increased notably during acetic acid fermentation (e.g., JR3: 7.61%; YO3: 0.98%) (Table S1).

### 3.3. Organic Acids

Organic acids, which contribute to the taste and functional characteristics of vinegar, vary in composition and concentration depending on the raw materials and fermentation conditions used during production [33]. The results for this stage of the study are shown in Table 2. Acetic acid, the primary organic acid in vinegar, was found to be present in the highest concentrations in all four types of grain vinegar at the end of fermentation. Among the vinegars, the five-grain vinegar (YO3), which had the highest acidity (14.67%), also contained the highest level of acetic acid, at 58.07 mg/mL. This was followed by rice vinegar (JR3), in which the level of acetic acid increased sharply from 2.53 mg/mL (JR1) to 42.99 mg/mL, representing approximately a 17-fold rise in acetic acid production. The barley vinegar (SB3) contained 36.99 mg/mL of acetic acid, while the brown rice vinegar (HB3), which had the lowest final acidity (4.87%), also had the lowest acetic acid concentration, at 13.59 mg/mL.

The levels of lactic acid, detected in all the samples, generally decreased as acetic acid fermentation progressed. This is likely due to the fermentation broth used in the process. Lactic acid levels should be minimized to ensure product standardization and safety in vinegar production [34]. A slight increase in lactic acid levels was observed in SB2 (20.00 mg/mL) and JR2 (12.75 mg/mL) during fermentation, possibly due to an insufficient nutrient supply for acetic acid production. In such conditions, lactic acid bacteria may convert sugars into lactic acid instead of acetic acid. Therefore, adjustments in the manufacturing process, such as increasing the inoculation volume of the starter culture to accelerate pH reduction or enhancing the oxygen supply to inhibit lactic acid bacteria, may be necessary. Oxygen exposure can produce reactive oxygen species that suppress the activity of these anaerobic bacteria [35].

The levels of malic acid, which was present in trace amounts before fermentation, completely depleted in some samples as fermentation progressed. In particular, malic acid was no longer detected in the SB and JR samples during the acetic acid fermentation stage. This appears to be closely related to the increase in lactic acid bacteria numbers in these samples, as *Lactobacillus* species became more dominant over time. This strongly suggests that malic acid was converted into lactic acid via malolactic fermentation [36]. These results are consistent with previous studies reporting that the metabolic activity of lactic acid bacteria can significantly influence the organic acid composition during fermentation [37].

Additionally, propionic acid was found to be present at levels of 2.07 mg/mL in SB3 and 2.38 mg/mL in JR3. This acid occurs naturally during vinegar fermentation and is known to contribute to vinegar’s flavor. Thus, these samples are likely to have nutty and complex flavor profiles, as propionic acid has been reported to be associated with smooth and nutty taste characteristics [38]. In contrast, propionic acid was not detected in HB and YO, wherein acetic acid appeared to be the predominant organic acid, resulting in fresh and clean flavor profiles [39]. These differences are likely influenced by both the fermentation environment and microbial community structure, indicating the potential for diverse flavor profile characteristics depending on the type of vinegar.

### 3.4. Free Amino Acids

**Figure 4:**
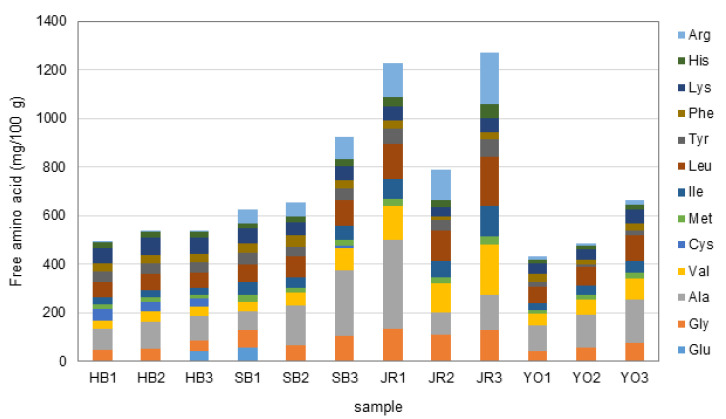
Free amino acids in grain vinegars produced via different fermentation processes across four regions and over three fermentation stages.

The free amino acids in vinegar contribute to the formation of various compounds during fermentation and ultimately influence flavor complexity and taste [40]. As shown in Figure 4 and Table S3, the free amino acids commonly detected at high levels during the fermentation of the four types of grain vinegar were those associated with sweet (alanine and glycine) and bitter (lysine, leucine, and valine) tastes. In particular, alanine was detected at especially high concentrations at the end of fermentation, namely, 100 mg/100 g in HB3, 269.43 mg/100 g in SB3, 145.87 mg/100 g in JR3, and 183.13 mg/100 g in YO3, potentially contributing to a sweeter sensory profile in these vinegars. Alanine, which can be synthesized from glucose and pyruvic acid, may be produced through microbial metabolism during fermentation, especially through the breakdown of carbohydrates and proteins abundant in grains such as rice and barley, as previously reported [41]. It is assumed that the activity of *L. acetotolerans*, which was dominant in the microbial community, and *A. pasteurianus*, which increased during acetic acid fermentation, contributes to the production of alanine.

In addition, bitter-tasting essential amino acids, lysine, leucine, and valine, were found in high concentrations in all four types of grain vinegars. Notably, in JR3, these amino acids were detected at levels of 57.86 mg/100 g, 205.16 mg/100 g, and 207.89 mg/100 g, respectively. As a result, JR3 exhibited the highest total free amino acid content among all the samples: 1270.58 mg/100 g. This difference can be attributed to the fact that rice, compared to other grains, is relatively rich in proteins and amino acids and contains little to no gluten, which facilitates the production of various free amino acids by acetic acid bacteria during fermentation, as previously suggested [41]. Among the cereal grains used in vinegar production, barley contains a relatively high proportion of gluten, whereas rice, brown rice, sorghum, millet, and foxtail millet are classified as low- or gluten-free grains. In particular, rice and sorghum are widely recognized as suitable ingredients for gluten-free diets [42,43]. Furthermore, essential amino acids such as histidine, phenylalanine, isoleucine, and methionine were also detected, suggesting these grains contribute to energy metabolism, immune function, and other physiological processes [44]. These results confirm the potential for further development based on the choice of raw materials and microbial activity, and we hope that these findings can be applied to improve vinegar quality and optimize production processes.

### 3.5. Volatile Flavor Components

**Figure 5:**
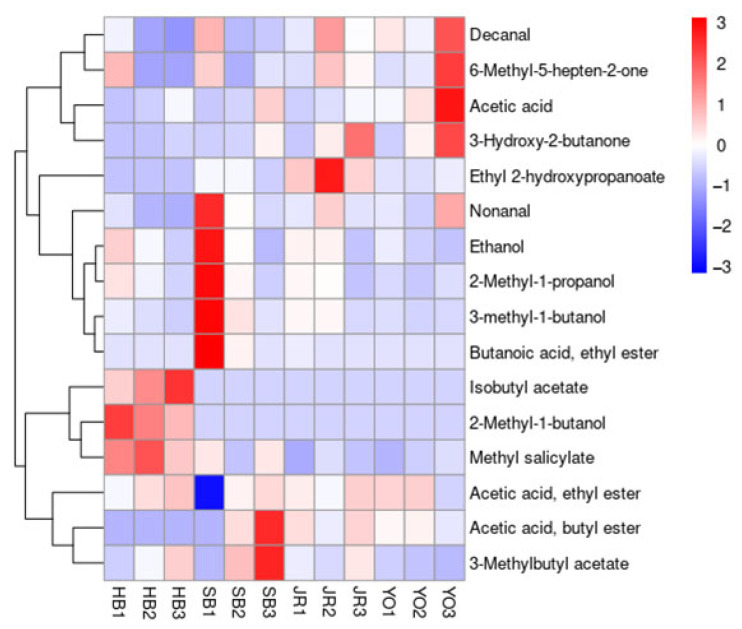
Heatmap of volatile flavor compounds in grain vinegars produced via different fermentation processes across four regions and over three fermentation stages.

The volatile flavor compounds of the grain vinegars were analyzed to identify aromatic substances, and their correlations among samples were visualized in a heatmap (Table 3, Figure 5). A total of 16 flavor compounds were detected and classified into alcohols, aldehydes, esters, acids, and ketones. As fermentation progressed, the concentration of alcohols (e.g., ethanol, 2-methyl-1-propanol, etc.) gradually decreased, as acetic acid bacteria consumed ethanol to produce acids through acetic fermentation. In addition to ethanol, aldehydes, such as nonanal and decanal, were also produced during fermentation. However, these aldehydes are more likely derived from the oxidative degradation of fatty acids rather than ethanol oxidation. They are known to contribute fresh, fruity, and natural aromas [45].

Esters, which are secondary metabolites formed through the reaction of alcohols and acids, increased in concentration over time, with six types detected [46]. Among them, acetic acid, ethyl ester was the most abundant, followed by 3-methylbutyl acetate and methyl salicylate, with particularly high levels observed in YO, which was produced from a multi-grain blend and had the highest acidity. According to previous studies, esters are the most influential class of flavor compounds, even though they are typically present in small amounts. Despite their low concentrations, they contribute significantly to flavor [47,48]. For instance, fruity and sweet aromas reminiscent of banana were detected in the grain vinegar, largely due to 3-methylbutyl acetate. This is consistent with earlier findings reporting that vinegars made from grains like rice, brown rice, and barley contain similar aroma-active compounds, which are mainly produced during microbial fermentation [49,50]. Acids (e.g., ethyl 2-hydroxypropanoate and acetic acid) and ketones (e.g., 3-hydroxy-2-butanone, 6-methyl-5-hepten-2-one, etc.) also contributed to the sweet and pleasant aromas and were found in higher concentrations in YO, reflecting the influence of this variety’s more diverse grain composition.

Based on these findings, we suggest that differences in aroma during vinegar fermentation are influenced by variations in the dominant microbial communities, which in turn are affected by the type of raw materials used. Previous studies on traditional Chinese grain vinegars have reported that environmental factors during fermentation significantly impact microbial dynamics and the production of key metabolites, including organic acids, amino acids, and volatile compounds. In particular, the acetic acid fermentation stage has been identified as a critical period for the accumulation of flavor compounds [51]. Accordingly, the results of this study are expected to serve as valuable foundational data for understanding how volatile flavor profiles in traditional grain vinegars are shaped by grain type and fermentation conditions.

## 4. Conclusions

In this study, traditional Korean grain vinegars were collected from different regions, and changes in microbial communities and their correlations with functional components during fermentation were monitored. Microbial community analysis revealed that *Lactobacillus acetotolerans* was the dominant species, while *Acetobacter pasteurianus* gradually increased in abundance during fermentation, playing an important role in acetic acid production. Specifically, *L. acetotolerans* is known for its resistance to acidic environments and contributes to increasing vinegar acidity, thereby enhancing its preservative properties. This property, along with the activity of *A. pasteurianus*, significantly contributed to the increase in functional components, particularly organic acids such as acetic acid, in the grain vinegars. Additionally, the production of free amino acids varied depending on the interaction between the fermentation mash and microbial communities, with alanine playing a key role in enhancing the sweetness of the vinegar at the end of fermentation. In contrast, volatile flavor compounds appeared to be more strongly influenced by the type of raw materials used than by the microbial community. Complex substrates, such as the five-grain vinegar (YO), tended to generate a greater variety and intensity of volatile compounds, suggesting that the unique chemical compositions of raw materials play a crucial role in the formation of these compounds during fermentation. These findings confirm that both raw material selection and fermentation conditions have a significant impact on the development of flavor components in grain vinegar and provide valuable foundational data for optimizing vinegar production processes to enhance both functionality and flavor.

## Figures and Tables

**Table 1:** Manufacturing conditions and acidity of collected grain vinegars.

Type of Sample	Region	Raw Material	Fermenter	Temperature	Period (Days)	Sample Code	Acidity (%)
HB	Gangwon state	Brown rice (100%)	Earthenware Jar	28–30 °C	0	HB1	1.60 ± 0.03 ^c 1^
					10	HB2	3.16 ± 0.01 ^b^
					20	HB3	4.87 ± 0.02 ^a^
SB	Gyeonggi-do	Barley (100%)	Glass	30 °C	0	SB1	1.41 ± 0.02 ^b^
					10	SB2	2.51 ± 0.02 ^a^
					20	SB3	6.83 ± 0.02 ^c^
JR	Jeonbuk state	Rice (100%)	Earthenware Jar	30 °C	0	JR1	1.22 ± 0.02 ^a^
					10	JR2	2.52 ± 0.01 ^a^
					20	JR3	5.43 ± 0.02 ^b^
YO	Gyeongsangbuk-do	Five-grain (brown rice (65%), barley (20%), sorghum (5%), millet (5%), and foxtail millet (5%))	Stainless Steel	30 °C	0	YO1	8.54 ± 0.02 ^d^
					10	YO2	9.53 ± 0.01 ^c^
					20	YO3	14.67 ± 0.01 ^d^

^1^ The values are means ± SD (*n* = 3); different letters within the same column indicate a significant difference (*p* < 0.05).

**Table 2:** Organic acid levels in grain vinegars produced via different fermentation processes across four regions and over three fermentation stages.

Sample	Organic Acid (mg/mL)						Total
	Citric Acid	Malic Acid	Succinic Acid	Lactic Acid	Acetic Acid	Propionic Acid	
HB1	N.D. ^1^	N.D.	N.D.	9.36 ± 0.80 ^abc^	1.66 ± 0.16 ^a^	N.D.	11.02 ± 0.96
HB2	N.D.	N.D.	N.D.	3.53 ± 0.33 ^ab^	6.04 ± 0.56 ^ab^	N.D.	9.56 ± 0.89
HB3	N.D.	N.D.	N.D.	0.15 ± 0.02 ^a^	13.59 ± 1.18 ^b^	N.D.	13.75 ± 1.20
SB1	0.63 ± 0.02 ^c 2^	0.15 ± 0.26 ^ab^	0.51 ± 0.00 ^ab^	16.5 ± 9.48 ^bc^	4.37 ± 3.35 ^a^	0.30 ± 0.22 ^a^	22.00 ± 13.33
SB2	0.27 ± 0.31 ^ab^	N.D.	1.08 ± 0.54 ^bcd^	20.00 ± 12.64 ^c^	9.33 ± 1.19 ^ab^	3.55 ± 1.69 ^bc^	34.23 ± 16.37
SB3	0.25 ± 0.30 ^ab^	N.D.	0.76 ± 0.41 ^bc^	10.23 ± 4.32 ^abc^	36.99 ± 11.83 ^d^	2.07 ± 3.58 ^abc^	50.30 ± 20.43
JR1	0.64 ± 0.04 ^c^	0.32 ± 0.28 ^c^	1.35 ± 0.73 ^cd^	10.59 ± 9.41 ^abc^	2.53 ± 3.51 ^a^	0.18 ± 0.24 ^a^	15.61 ± 14.22
JR2	0.44 ± 0.32 ^bc^	N.D.	1.48 ± 0.37 ^d^	12.75 ± 12.58 ^abc^	9.85 ± 0.87 ^ab^	2.57 ± 1.78 ^abc^	27.10 ± 15.91
JR3	0.44 ± 0.32 ^bc^	N.D.	1.04 ± 0.45 ^bcd^	7.88 ± 4.18 ^abc^	42.99 ± 10.55 ^d^	4.38 ± 3.79 ^c^	56.72 ± 19.29
YO1	1.48 ± 0.01 ^d^	N.D.	0.87 ± 0.01 ^bcd^	5.93 ± 0.09 ^ab^	25.88 ± 0.70 ^c^	0.95 ± 0.12 ^ab^	35.11 ± 0.92
YO2	1.39 ± 0.00 ^d^	N.D.	0.86 ± 0.00 ^bcd^	5.96 ± 0.09 ^ab^	40.04 ± 0.34 ^d^	N.D.	48.25 ± 0.44
YO3	1.45 ± 0.01 ^d^	N.D.	0.83 ± 0.00 ^bc^	4.48 ± 2.43 ^ab^	58.07 ± 0.31 ^e^	N.D.	64.84 ± 2.75

^1^ N.D., not detected. ^2^ The values are means ± SD (*n* = 3); different letters within the same column indicate a significant difference (*p* < 0.05).

**Table 3:** Volatile flavor compounds in grain vinegars produced using different fermentation processes across four regions.

Compound	Order Description	Type of Sample							
		HB		SB		JR		YO	
		*p*-Value ^1^	VIP ^2^	*p*-Value	VIP	*p*-Value	VIP	*p*-Value	VIP
Alcohols									
Ethanol	Alcohol	2.85 × 10^−3^	1.02	2.78 × 10^−6^	0.96	3.66 × 10^−6^	0.95	1.33 × 10^−7^	1.01
2-Methyl-1-propanol	Nail-polish-like, sweet	2.01 × 10^−2^	0.89	3.25 × 10^−6^	0.97	3.09 × 10^−7^	1.01	2.06 × 10^−7^	1.02
2-Methyl-1-butanol	Sweet, fruity	3.92 × 10^−1^	0.99	N.D.	N.D.	N.D.	N.D.	N.D.	N.D.
3-Methyl-1-butanol	Rancid, banana-like	2.60 × 10^−2^	0.90	1.11 × 10^−5^	0.95	7.00 × 10^−9^	1.03	4.55 × 10^−9^	1.02
Alehydes									
Nonanal	Sweet orange, rose	5.83 × 10^−2^	1.02	2.59 × 10^−1^	1.05	1.21 × 10^−2^	0.68	2.36 × 10^−1^	0.73
Decanal	Grass-like	6.53 × 10^−2^	0.77	1.00	0.04	2.21 × 10^−1^	0.96	1.99 × 10^−1^	0.43
Esters									
Acetic acid, ethyl ester	Sweet, soapy, grass-like	7.39 × 10^−6^	1.16	4.53 × 10^−6^	1.08	4.36 × 10^−9^	1.01	2.05 × 10^−8^	1.30
Acetic acid, butyl ester	Banana-like, appley, sweet	N.D.	N.D.	5.91 × 10^−5^	1.01	8.94 × 10^−4^	0.89	1.52 × 10^−3^	1.21
Isobutyl acetate	Pear, apple	5.76 × 10^−5^	0.99	N.D.	N.D.	N.D.	N.D.	N.D.	N.D.
Butanoic acid, ethyl ester	Fruity, pineapple-like	N.D.	N.D.	2.50 × 10^−7^	0.91	6.79 × 10^−7^	1.56	N.D.	N.D.
3-Methylbutyl acetate	Banana-like	6.04 × 10^−5^	0.99	8.61 × 10^−7^	1.04	4.81 × 10^−8^	0.89	2.59 × 10^−5^	1.24
Methyl salicylate	Sweet, fruity	7.38 × 10^−2^	1.33	7.41 × 10^−2^	0.72	2.92 × 10^−3^	1.20	8.17 × 10^−4^	1.12
Acids									
Ethyl 2-hydroxypropanoate	Sweet, fruity	N.D.	N.D.	5.29 × 10^−5^	1.16	3.34 × 10^−7^	0.65	1.55 × 10^−2^	0.84
Acetic acid	Pungent	6.34 × 10^−3^	0.98	1.55 × 10^−7^	1.14	7.20 × 10^−8^	0.95	2.24 × 10^−7^	1.02
Ketones									
3-Hydroxy-2-butanone	Buttery, creamy	8.08 × 10^−4^	1.18	7.68 × 10^−8^	1.21	1.76 × 10^−10^	1.01	6.04 × 10^−9^	1.02
6-Methyl-5-hepten-2-one	Herby, grass-like	5.02 × 10^−1^	0.54	3.10 × 10^−1^	1.18	2.39 × 10^−1^	0.89	2.01 × 10^−1^	0.68

^1^ *p*-values (*p* < 0.05) were analyzed using one-way analysis of variance (ANOVA). ^2^ Variable importance in the projection (VIP > 1.0) values was determined using partial least squares discriminant analysis (PLS-DA). N.D., not detected.

## Data Availability

The original contributions presented in this study are included in the article/Supplementary Material. Further inquiries can be directed to the corresponding author.

## Supplementary Materials

The following supporting information can be downloaded at https://www.mdpi.com/article/10.3390/microorganisms13061308/s1. Table S1: Microbial communities of grain vinegars according to production process; Table S2: Richness and α-diversity (Shannon, Simpson) of grain vinegars according to production process; Table S3: Free amino acids of grain vinegars according to production process.
