# Supplementary material for: Microbial Community and Functional Analysis of Regionally Produced Traditional Korean Grain Vinegar
Source: Microorganisms. 2025 Jun 4;13(6):1308. doi: 10.3390/microorganisms13061308 (PMC12195155; doi:10.3390/microorganisms13061308)
Supplement: Supplementary file 1 [file microorganisms-13-01308-s001.zip › microorganisms-3609816-supplementary.pdf]

Microbial Community and Functional Analysis of Regionally Produced Traditional Korean Grain Vinegar

Su Jeong Lee, Sun Hee Kim, Hee-Min Gwon, Jinju Park *

Fermented and Processed Food Research Division, Department of Food Sciences, NICS, RDA, Wanju, 55365, Republic of Korea

Supplementary files:

Table S1. Microbial communities in grain vinegars produced by different fermentation processes across four regions and three fermentation stages.

Table S2. Richness and α-diversity (Shannon, Simpson) in grain vinegars produced by different fermentation processes across four regions and three fermentation stages.

Table S3. Free amino acids in grain vinegars produced by different fermentation processes across four regions and three fermentation stages.

Table S1. Microbial communities in grain vinegars produced by different fermentation processes across four regions and three fermentation stages.

	HB1	HB2	HB3	SB1	SB2	SB3	JR1	JR2	JR3	YO1	YO2	YO3
L.ac. [1]	47.26	48.98	48.60	59.89	75.13	88.29	97.68	99.61	91.94	88.15	98.15	97.19
L.cu.	0.03	0.06	0.10	25.19	11.20	0.85	0.20	0.07	0.08	N.D.	N.D.	N.D.
A.pa.	0.05	36.50	35.55	N.D.	0.93	9.81	0.15	0.13	7.61	0.03	0.02	0.98
S.ce.	1.76	0.39	0.41	0.13	0.01	N.D.	0.17	0.01	0.00	0.02	N.D.	N.D.
L.fe.	N.D.	N.D.	N.D.	0.04	0.05	0.01	0.09	0.01	0.02	11.51	1.76	1.78
P.pe.	N.D.	N.D.	N.D.	0.11	0.16	0.01	0.03	0.00	0.01	N.D.	N.D.	N.D.
L.ci.	0.01	0.00	0.01	5.32	2.03	0.12	0.06	0.02	0.05	0.02	0.00	0.00
W.ci.	N.D.	N.D.	N.D.	0.26	3.90	0.24	0.00	N.D.	0.00	0.22	0.05	0.03
L.co.	N.D.	N.D.	N.D.	0.01	0.01	N.D.	0.02	0.01	0.01	N.D.	N.D.	N.D.
L.ho.	N.D.	N.D.	N.D.	3.44	2.37	0.20	N.D.	N.D.	N.D.	0.00	N.D.	N.D.
L.la.	N.D.	N.D.	N.D.	1.39	2.49	0.31	0.01	0.00	0.01	0.01	N.D.	N.D.
L.pl.	N.D.	N.D.	N.D.	0.04	0.07	0.01	0.05	0.00	0.02	N.D.	N.D.	N.D.
C.cr.	N.D.	N.D.	N.D.	0.01	0.02	0.00	0.00	N.D.	0.00	N.D.	N.D.	N.D.
L.pa.	37.48	6.46	13.63	0.00	0.00	0.03	1.47	0.13	0.21	N.D.	N.D.	0.00
K.co.	0.01	0.00	N.D.	0.11	0.14	0.02	0.00	N.D.	0.00	0.00	N.D.	N.D.
L.hi.	7.70	2.94	1.46	N.D.	N.D.	N.D.	N.D.	N.D.	N.D.	N.D.	N.D.	N.D.
L.pe.	5.33	4.30	0.20	N.D.	N.D.	N.D.	N.D.	N.D.	N.D.	N.D.	N.D.	N.D.
L.ha.	0.02	0.29	N.D.	N.D.	N.D.	N.D.	N.D.	N.D.	N.D.	N.D.	N.D.	N.D.
L.bu.	0.00	0.00	N.D.	N.D.	N.D.	N.D.	N.D.	N.D.	N.D.	N.D.	N.D.	N.D.
B.ve.	0.28	0.05	0.03	N.D.	N.D.	N.D.	N.D.	N.D.	N.D.	N.D.	N.D.	N.D.
L.me.	0.01	0.00	0.00	N.D.	N.D.	N.D.	N.D.	N.D.	N.D.	N.D.	N.D.	N.D.
Other	0.15	0.03	0.01	1.17	1.49	0.11	0.07	0.01	0.03	0.05	0.02	0.02

[1] *L.ac., Lactobacillus acetotolerans; L.cu., Latilactobacillus curvatus; A.pa., Acetobacter pasteurianus;. S.ce., Saccharomyces cerevisiae; L.fe., Limosilactobacillus fermentum; P.pe., Pediococcus pentosaceus; L.ci., Leuconostoc citreum; W.ci., Weissella civaria; L.co.; Lactobacillus coryniformis; L.ho.; Leuconostoc holzapfelii; L.la., Latiplantibacillus plantarum; C.cu., Companilactobacillus crustorum; L.pa., Lacticaseibacillus paracasei; K.co., Kosakonia cowanii; L.hi., Lentilactobacillus hilgardii; L.pe., Lactobacillus perolens; L.ha., Lactobacillus harbinensis; L.bu., Lentilactobacillus buchneri; B.ve., Bacillus velenzensis; L.me., Leuconostoc mesenteroides;* Other, *Levilactobacillus brevis; Leuconostoc pseudomesenteroides; Companilactobacillus paralimentarius; Leuconostoc lactis.* [2] N.D., not detected.

Table S2. Richness and α-diversity (Shannon, Simpson) in grain vinegars produced by different fermentation processes across four regions and three fermentation stages.

	Richness	Shannon	Simpson
HB1	26	1.186001	0.628033
HB2	19	1.183352	0.619952
HB3	13	1.097825	0.618598
SB1	41	1.197429	0.572589
SB2	45	1.013884	0.419729
SB3	23	0.446854	0.210787
JR1	23	0.143909	0.045674
JR2	16	0.031377	0.007694
JR3	20	0.304953	0.148886
YO1	19	0.383942	0.209745
YO2	7	0.096153	0.036270
YO3	10	0.149014	0.055021

Table S3. Free amino acids in grain vinegars produced by different fermentation processes across four regions and three fermentation stages.

	HB1	HB2	HB3	SB1	SB2	SB3	JR1	JR2	JR3	YO1	YO2	YO3
Glu [1]	3.9	5.1	43.6	57.4	N.D.[2]	N.D.	N.D.	N.D.	N.D.	N.D.	N.D.	N.D.
Gly	41.8	47.1	39.1	73.3	67.7	103.0	135.4	110.7	128.5	43.8	55.3	72.9
Ala	86.9	110.5	101.5	74.6	159.6	269.4	365.2	87.9	145.9	103.1	136.6	183.1
Val	36.2	44.8	40.3	39.2	55.6	91.5	139.3	121.7	207.9	46.8	62.1	82.4
Cys	47.5	37.3	33.2	N.D.	N.D.	10.4	N.D.	N.D.	N.D.	N.D.	N.D.	N.D.
Met	18.1	18.2	17.3	26.4	20.0	25.7	29.0	24.7	33.4	18.8	19.8	25.6
Ilu	27.1	27.7	28.5	54.9	40.7	59.4	82.0	69.7	122.5	26.8	37.4	50.4
Leu	63.4	67.1	61.4	74.2	87.3	104.7	146.2	122.5	205.2	67.9	79.6	106.4
Tyr	44.5	44.6	45.3	44.6	40.5	45.9	59.5	44.0	72.4	18.1	9.1	18.0
Phe	32.5	34.4	33.7	42.1	48.5	34.0	34.4	13.4	28.5	33.4	19.2	29.6
Lys	62.7	72.4	65.0	62.32	53.7	58.6	60.7	41.3	57.9	46.6	41.6	56.8
His	24.9	26.3	23.6	16.3	20.8	30.6	36.7	30.4	59.5	14.9	15.8	21.5
Arg	0.9	0.9	3.2	57.9	62.1	89.3	140.0	120.9	209.0	11.1	10.4	15.9
Tatal	490.4	536.2	535.4	623.0	656.5	922.6	1228.4	787.1	1270.6	431.3	486.9	662.6

[1] Glu, Glutamic acid; Gly, Glycine; Ala, Alanine; Val, Valine; Cys, Cystine; Met, Methionine; Ile, Isoleucine; Leu, Leucine; Tyr, Tyrosine; Phe, Phenylalanine; Lys, Lysine; His, Histidine; Arg, Arginine. [2] N.D., not detected.
